# Budget Impact Analysis of *EGFR* Mutation Liquid Biopsy for First- and Second-Line Treatment of Metastatic Non-Small Cell Lung Cancer in Greece

**DOI:** 10.3390/diagnostics10060429

**Published:** 2020-06-24

**Authors:** Mindy Cheng, Athanasios Akalestos, Sidney Scudder

**Affiliations:** 1 Roche Molecular Systems, Inc., Pleasanton, CA 94588, USA; 2 Roche Diagnostics (Hellas) S.A., 151 25 Marousi, Greece; thanasis.akalestos@roche.com; 3 Roche Sequencing Solutions, Pleasanton, CA 94588, USA; sid.scudder@roche.com

**Keywords:** budget impact, cost, epidermal growth factor receptor (*EGFR*), plasma biopsy, non-small cell lung cancer (NSCLC), companion diagnostic

## Abstract

Within the European Union, Greece has the highest incidence of lung cancer among people under 45 years of age. Epidermal growth factor receptor (*EGFR*) tyrosine kinase inhibitors are indicated for the treatment of patients with *EGFR* mutation-positive metastatic non-small cell lung cancer (mNSCLC). Tumor tissue biopsy is the standard method for *EGFR* mutation detection but is invasive, is resource-intensive, and has risks of complications. The objective of this analysis was to estimate the financial impact on the Greek National Health System of adopting plasma biopsy and to identify the cost-optimal approach for *EGFR* mutation testing of patients with mNSCLC. We developed a budget impact model to estimate total costs for three *EGFR* mutation testing approaches: (1) plasma test, (2) combined testing (tissue and plasma test), and (3) reflex testing, compared to the current scenario of tissue biopsy only. One-way sensitivity and scenario analyses were conducted to evaluate the impact of uncertainty and variance of different input parameters on the results. In the first-line (1L) setting, base-case results showed that adopting plasma testing in a combined testing approach identified more *EGFR* mutation-positive patients and yielded cost savings (−€17 per correctly classified patient) relative to tissue testing alone. The reflex testing approach was the cost-optimal strategy in the second-line (2L) setting as it identified the most *EGFR* mutation-positive patients with cost savings of −€42 per correctly classified patient relative to tissue testing alone. This analysis suggests that access to both *EGFR* mutation tissue and plasma testing are important for optimizing mNSCLC treatment decisions in Greece. Inclusion of plasma testing in either a combined or reflex testing approach may be cost optimal for *EGFR* mutation plasma test implementation.

## 1. Introduction

Lung cancer is one of the most commonly diagnosed carcinomas globally and accounts for approximately 15% of all cancers in men and 8% among women [1]. Lung cancer accounts for a large proportion (18%) of all cancer-related deaths worldwide which equates to over 1.7 million deaths in 2018 [1,2]. Higher lung cancer prevalence and mortality rates are observed among men in Southern Europe, East Asia, South Africa, and the Far East. Within the European Union (EU), Hungary and Serbia have the highest lung cancer mortality rates, while Greece has the highest incidence of lung cancer among people under 45 years of age, attributed to early exposure to cigarettes and smoking [3].

Approximately 85–90% of all lung cancer cases are non-small cell lung cancer (NSCLC), with the majority of patients presenting with advanced or metastatic disease at the time of diagnosis [4,5]. Activating mutations in exons 19 or 21 of the tyrosine kinase domain of the epidermal growth factor receptor (*EGFR*) gene are present in 12–40% of non-small cell lung cancer (NSCLC) tumors and have been demonstrated to predict clinical response to first- and second-line *EGFR* tyrosine kinase inhibitors (TKIs) with differentiated outcomes (response rates, progression-free survival, and quality of life) compared to standard chemotherapy [6,7,8,9,10,11,12,13,14]. Current practice recommendations by the International Association for the Study of Lung Cancer (IASL) and the European Society for Medical Oncology (ESMO), among others, are molecular diagnostic testing to identify patients diagnosed with advanced NSCLC eligible for first- or second-line *EGFR* TKI targeted therapy [15,16].

The most common methods of obtaining lung tumor tissue for molecular testing are via bronchoscopy by an expert operator or via fine needle aspirate/core needle biopsy depending upon the location of the tumor in the lungs and on the fitness of the patient. Lung tumor biopsy is associated with numerous challenges including procedural invasiveness with risk of adverse event (e.g., pneumothorax), logistical complications, sample availability, and tumor heterogeneity. Patients with low performance status or comorbidities may be ineligible to undergo lung biopsy procedures. Tumors may also occur in tissues that are difficult to access with biopsy or may be of limited size for adequate sampling. Additionally, malignant tumors have significant molecular heterogeneity that may require sampling from multiple locations to obtain a comprehensive molecular profile [17]. In the landmark IPASS trial, it was observed that approximately 58% of patients was unable to undergo tissue biopsy or did not provide samples that could be successfully evaluated [9]. In another multi-center, prospective, pragmatic study that examined the feasibility of re-biopsy among patients with advanced NSCLC at disease progression, researchers observed that re-biopsy could not be performed on 18% of patients mainly because of anticoagulation. Among eighty-two patients who underwent re-biopsy, approximately 26% of the samples contained insufficient tumor cells for molecular analysis [18]. Post-biopsy procedural complication rates have been reported from 0–54%, primarily pneumothorax requiring chest tube drainage, hemoptysis, and intrapulmonary hemorrhage [18,19,20,21]. The challenges and barriers associated with conventional tissue biopsy may lead to treatment delays or uncertain treatment decisions when molecular testing results cannot be obtained.

*EGFR* mutations identified in blood circulating cell-free DNA (cfDNA) have been shown to similarly predict response to *EGFR* tyrosine kinase inhibitors in the 1L and 2L treatments of mNSCLC with comparable accuracy to mutations identified via tumor tissue biopsy [22]. As such, new molecular testing modalities using cfDNA have emerged (“liquid biopsy”) that overcome many of the significant barriers associated with conventional tumor tissue biopsy such as the following:

- Liquid biopsy offers a minimally invasive method for molecular diagnostic testing with lower patient burden relative to tissue biopsy.
- Liquid biopsy enables more timely and frequent *EGFR* mutation assessment due to lower logistical burdens with a blood draw procedure compared to an invasive surgical procedure.
- Liquid biopsy enables all patients with NSCLC the opportunity to undergo *EGFR* mutation testing for optimal therapy selection.

As the number of predictive gene biomarkers and targeted therapies for NSCLC continues to grow, there is also increasing evidence demonstrating the clinical utility and cost-effectiveness of next-generation sequencing (NGS) multiplex panels, compared to sequential testing of individual gene targets (e.g., *EGFR, ALK, BRAF*, etc.). Despite the growing body of evidence supporting the use of NGS to inform NSCLC treatment decisions, access to NGS in clinical settings for routine molecular oncology testing remains limited in many countries, possibly due to affordability challenges associated with higher costs of NGS technologies. Specifically, in Greece, NGS would add complexity to the sample workflow as pathology laboratories do not perform NGS. If NGS is to be performed, the specimen would have to be transferred from a pathology laboratory to a molecular laboratory, which is usually located in a separate facility. In these settings where NGS has not been widely adopted, single gene testing for *EGFR* mutation remains the standard of care for patients diagnosed with NSCLC. Currently, the Greek National Health System provides access to *EGFR* mutation testing for patients diagnosed with NSCLC but only from tumor tissue specimens. The objective of this analysis is to estimate the financial impact on the Greek National Health System of adopting an alternative, less invasive testing option of plasma-based *EGFR* mutation testing and to identify the most cost-optimal *EGFR* mutation testing approach to support treatment of patients diagnosed with NSCLC in Greece.

## 2. Materials and Methods

### 2.1. Model Overview

A budget impact model (BIM) was developed with Microsoft^®^ Excel 2010 and uses a population-based approach to compare the financial impact of four potential *EGFR* mutation testing strategies. This was accomplished by first estimating the base-line financial impact for the “current scenario” where eligible patients diagnosed with metastatic NSCLC (mNSCLC) were assumed to have access to a lung tissue biopsy procedure (standard of care). This was followed by estimating the financial impact for “projected scenarios” that incorporated *EGFR* plasma testing (liquid biopsy) via three different potential approaches:

- plasma testing as an alternative to tissue biopsy for all (“plasma test only”);
- primary tissue testing with plasma testing only for patients who are ineligible for tissue biopsy (“combined testing approach”); and
- primary plasma testing for all with reflex of wild-type patients to confirmatory tissue biopsy (“reflex testing approach” per U.S. Food and Drug Administration (FDA)-approved intended use for the cobas^®^ *EGFR* Mutation Test v2, Roche Molecular Systems, Pleasanton, CA, USA [23]. Due to lower test concordance between plasma and tissue testing, it is recommended that patients who are *EGFR* mutation negative by plasma undergo confirmatory tissue testing when practicable.)

The budget impact (BI) is calculated as the financial difference between a “projected scenario” and “current scenario”.

As there are numerous *EGFR* mutation tests, both regulatory approved commercial assays and laboratory-developed tests, we based this analysis on the cobas^®^ *EGFR* Mutation Test v2 due to available published data. The cobas^®^ *EGFR* Mutation Test v2 was the first regulatory approved (U.S. FDA, CE-IVD) genetic test for the qualitative detection and identification of 42 defined mutations of the *EGFR* gene from DNA derived from formalin-fixed paraffin-embedded tumor tissue (FFPET) or cfDNA derived from plasma of patients with NSCLC; it is the only commercial test system capable of testing for *EGFR* mutations from FFPET and plasma specimens at the same time (mixed sample type capability). The test is indicated as a companion diagnostic to aid in selecting NSCLC patients for treatment with the targeted therapies erlotinib (first-line therapy; exon 19 deletions and L858R) and osimertinib (second-line therapy; T790M resistance mutation) [23]. Although the test may be used for people diagnosed with NSCLC for which *EGFR* mutation testing is indicated, plasma testing may provide the greatest benefit to those who are too ill to undergo tissue biopsy or are otherwise unable to provide a tumor tissue specimen for *EGFR* mutation testing. We incorporated test performance estimates for the cobas^®^ *EGFR* Mutation Test v2 to calculate the BI and to report results on the basis of each mNSCLC patient expected with a correctly classified *EGFR* mutation status and separately on the basis of each diagnosed mNSCLC patient for comparative purposes.

### 2.2. Population

In the base-case analysis, the model applies the age-standardized country-specific annual incidence of lung cancer to the 2018 general population in Greece to mathematically project the number of patients expected to be diagnosed with lung cancer in a given year. A series of epidemiological estimates derived from the published literature specific to the Greek population, when available, were subsequently applied to approximate the number of patients diagnosed with mNSCLC (adenocarcinoma, large cell, or unspecified histology) for which tumor molecular profiling is recommended to guide first-line therapy decisions [15,24]. Estimates derived from the published literature and clinical opinion (when estimates were not identified in the public domain) were applied to the newly diagnosed mNSCLC population to project the number of patients eligible for *EGFR* mutation testing via lung tissue biopsy. It was assumed that all patients could be eligible for a blood draw for plasma biopsy. Separately, estimates were applied to the cohort of patients assumed treated with a first-line (1L) anti-*EGFR* therapy (e.g., erlotinib) to project the cohort eligible for tissue re-biopsy at disease progression and second-line (2L) therapy (e.g., osimertinib) at any future time. The prevalence of *EGFR* mutations among newly diagnosed patients and specifically T790M resistance mutations among patients with tumor progression was used to project the number of patients expected to test *EGFR* mutation-positive in order to estimate costs of a reflex testing approach. The epidemiological estimates used to project the eligible testing populations are described in Table 1.

### 2.3. Clinical Inputs

In the base-case analysis, the test performance data (sensitivity and specificity) for tissue samples (exons 19 and 21) for newly diagnosed cases in the 1L setting were derived from a randomized phase III clinical trial (EURTAC) that assessed the safety and efficacy of erlotinib compared with standard chemotherapy as a first-line treatment for European patients with advanced *EGFR* mutation-positive NSCLC in which the clinical validity of the cobas^®^ *EGFR* Mutation Test was evaluated [32]. The test performance estimates for plasma samples, exons 19 and 21 at diagnosis and T790M resistance mutation at disease progression, were derived from the clinical utility study that evaluated the cobas^®^ *EGFR* Mutation Test v2 for detection of *EGFR* T790M mutation in patients with advanced NSCLC [33]. A recent systematic review and meta-analysis evaluated the diagnostic accuracy of the pooled category of cfDNA (plasma) tests for the detection of *EGFR* T790M mutation among NSCLC patients who progressed after *EGFR*-TKIs [34]. Since the systematic review suggested significantly lower test performance of the pooled category of *EGFR* plasma tests compared to the test performance observed for the cobas^®^ *EGFR* Mutation Test v2 individually, we conducted a scenario analysis using the test performance data from the systematic review to evaluate the impact of lower test performance on results. In the base-case analysis, the proportion of patients assumed to experience an adverse event or complication from tissue biopsy, assumed pneumothorax requiring medical intervention, was derived by taking the average between two studies that evaluated complication rates after transthoracic needle biopsy or bronchoscopy procedures [20,21]. The clinical inputs are described in Table 2.

### 2.4. Cost Inputs

The costs included in this analysis reflect the Greek National Health System procedural reimbursement rates as of December 2018. The total cost of each testing approach was calculated by summing the cost of sample collection (tissue biopsy procedure or blood draw), the cost to treat biopsy complications (assumed pneumothorax requiring medical intervention), and the procedural reimbursement for a laboratory to perform an *EGFR* mutation test. Currently, the National Health System of Greece provides access to *EGFR* mutation tests only for tissue specimens. For the purpose of this analysis, we assumed the same reimbursement rate for *EGFR* mutation tests with plasma specimens (liquid biopsy) as with tissue specimens. The cost inputs used in the base-case analysis are described in Table 3.

### 2.5. One-Way Sensitivity Analysis

One-way sensitivity analysis (OWSA) was conducted on all model inputs to evaluate the impact of uncertainty on results. Each clinical and cost parameter was independently varied ±20% while keeping other parameters constant.

## 3. Results

### 3.1. Base-Case Analysis

Using the referenced data inputs, in the 1L setting, it was estimated that adopting plasma testing in a combined or reflex testing approach enabled more patients to undergo molecular diagnostic testing and identified more *EGFR* mutation-positive patients than tissue testing or plasma testing alone. The combined testing approach of primary tissue testing and plasma testing only for patients who are ineligible for tissue biopsy was cost saving relative to tissue testing alone on the basis of each correctly classified patient (−€17). On the basis of each diagnosed mNSCLC patient, the combined testing approach resulted in BI on the Greek National Health System of €22 relative to tissue testing alone. In this scenario, the BI is attributed to additional plasma tests for patients previously ineligible for tissue biopsy and therefore a larger patient population undergoing *EGFR* mutation testing. The reflex testing approach was the cost-optimal strategy in the 2L setting as it identified the greatest number of *EGFR* mutation-positive patients eligible for targeted therapy and yielded −€42 cost savings per correctly classified patient. Table 4 presents the results of the base-case analysis.

### 3.2. One-Way Sensitivity Analysis

In OWSA of the combined testing approach in the 1L setting, results were most sensitive to variation in the procedural reimbursement cost of performing an *EGFR* mutation tissue test, the plasma test, and to the specificity of the tissue and plasma tests (exons 19 and 21). The procedural reimbursement cost of performing an *EGFR* mutation plasma test was assumed equivalent to a tissue test in the base-case scenario (€160). When the cost of an *EGFR* mutation plasma test was varied between €128 to €192, the cost savings associated with a combined testing approach varied between €21.60 and €11.80, respectively, per correctly classified patient. When the procedural cost of an *EGFR* tissue test was varied over the same range, the cost savings associated with a combined testing approach varied between €12.10 and €21.60, respectively, per correctly classified patient. This suggests that, if an *EGFR* mutation tissue test is more costly than an *EGFR* mutation plasma test, implementing a combined testing approach (plasma testing for patients who are ineligible for tissue testing) in the 1L setting could yield greater cost savings than tissue testing only. Similar results were observed with test specificity such that, when the plasma test specificity was varied between 75% and 100%, the cost savings varied between €10 and €19, respectively, per correctly classified patient. This suggests that, if plasma test specificity (exons 19 and 21) is higher than tissue test specificity, a combined testing approach in the 1L setting may yield greater cost-savings than tissue testing only.

**Figure 1:**
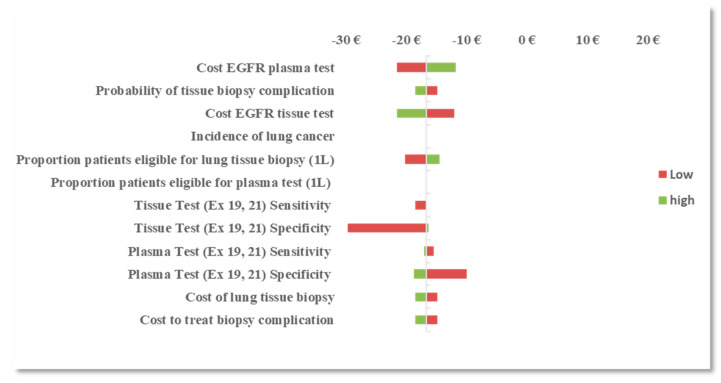
One-way sensitivity analysis (OWSA): difference in budget impact (BI) between the *EGFR* mutation combined testing approach and the tissue testing approach in the first-line (1L) setting.

**Figure 2:**
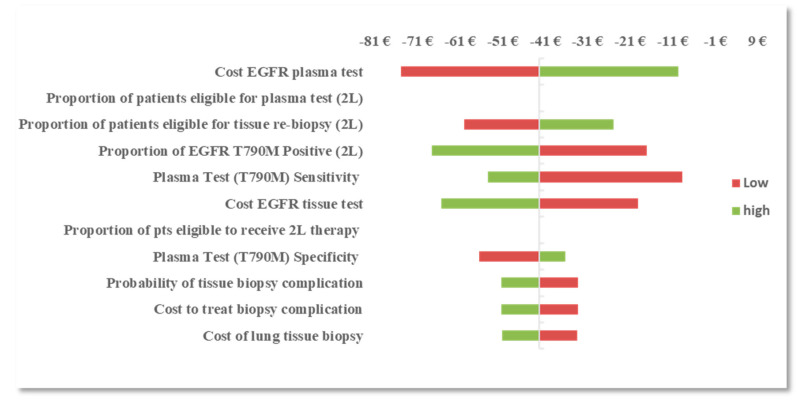
OWSA: difference in BI between the *EGFR* mutation plasma reflex testing approach and the tissue testing approach in the second-line (2L) setting.

With the reflex testing approach in the 2L setting, the procedural reimbursement cost of an *EGFR* mutation plasma test and tissue test remained the most influential parameters followed by the prevalence of T790M mutation at progression and plasma test sensitivity (T790M). With a reflex testing approach, the number of patients that are reflexed to tissue testing is dependent on the prevalence of T790M and the ability of the plasma test to identify true positives (sensitivity). As more patients are identified with T790M mutation, fewer patients are reflexed to tissue biopsy, therefore reducing the burden of tissue testing and the risk of complications and subsequent treatment costs. OWSA results are presented in Figure 1 and Figure 2.

### 3.3. Scenario Analysis

In order to address the differences between varying test performance across *EGFR* cfDNA (plasma) tests, a scenario analysis was conducted to evaluate the impact of lower test performance on the results. In the scenario analysis, we assumed a plasma test for the identification of T790M with 67% sensitivity and 80% specificity. Despite the lower test performance, the reflex testing approach remained the cost-optimal approach in the 2L setting as it identified the most *EGFR* T790M mutation-positive patients and was projected to yield cost savings of −€4 per correctly classified patient relative to tissue testing only. Compared to the base-case results, the total number of correctly classified patients in this scenario analysis decreased due to lower test performance inputs; however, a reflex testing approach incorporating the *EGFR* mutation plasma test was still projected to be beneficial toward enabling a larger population of patients to undergo molecular testing while reducing the burden of invasive tissue biopsy and associated risk of complications relative to the current scenario of tissue biopsy only. Table 5 presents results of this scenario analysis.

## 4. Discussion

Currently, the National Health System in Greece provides access to *EGFR* mutation testing for lung cancer patients via tissue biopsy. This study was not intended to evaluate liquid biopsy as a surrogate for histopathologic analysis. Histopathologic analysis to establish lung cancer diagnosis remains the gold standard and provides important diagnostic and prognostic information such as tumor subtype and staging. In this study, we developed a budget impact model to assess the population and financial impact on the Greek National Health System of adopting liquid biopsy as a less invasive, alternative molecular diagnostic approach to guide treatment decisions for patients newly diagnosed with NSCLC. As expected, compared to the current scenario of tissue testing only, the study showed that implementing *EGFR* mutation plasma testing overall would enable more lung cancer patients in Greece to undergo molecular diagnostic testing to guide therapy decisions, including patients with comorbid medical conditions or who are in extremis that preclude tissue biopsy. The findings suggest that implementing *EGFR* mutation plasma testing in the 1L setting via a combined testing approach and with a reflex testing approach in the 2L setting may yield cost savings. Due to lower test concordance between the plasma and tissue specimens for the T790M resistance mutation, likely due to tumor heterogeneity of T790M-mediated resistance, the reflex testing approach is recommended in the 2L setting in order to mitigate risk of false negative plasma results. The cost savings to a healthcare system associated with plasma testing implemented in either a combined or reflex testing approach are largely due to lower total costs of plasma testing relative to tissue biopsy and avoidance of potential tissue biopsy complications.

As we have previously suggested, all molecular diagnostic tests are not created equal and there are significant clinical and economic consequences to inaccurate *EGFR* mutation test results attributed to incorrect treatment decisions [38]. In addition to evaluating how best to implement new technologies, it is also important to understand the quality of technology offerings by assessing supporting bodies of evidence demonstrating analytical and clinical validity, and clinical utility. Toward the end of maximizing patient outcomes and of minimizing inefficiencies and medical waste, priority should be placed on adopting diagnostic tests with demonstrated test performance and clinical utility. For this reason, we felt it important to incorporate test performance in the analysis and to report results on the basis of correct *EGFR* mutation classification. We note that, in the 1L setting, the BI of a combined testing approach on the basis of a correctly classified patient suggests cost savings (−€17) relative to tissue testing alone whereas the same testing approach results in a budget impact (€22) on the basis of a mNSCLC patient. This may be explained by observing that the higher total cost of a combined testing approach is associated with a larger number of correctly classified patients relative to tissue testing alone. Therefore, the incremental costs associated with the combined testing approach are divided among a larger number of patients compared to the tissue testing approach leading to projected cost savings. Whereas on the basis of each mNSCLC patient, the incremental costs associated with the combined testing approach are divided among the same fixed number of newly diagnosed mNSCLC patients, resulting in a budget impact; this result is challenging to interpret as it includes both correct and incorrect test results. If the purpose of companion diagnostic tests is to select the correct patient for correct therapy, then it is important to understand the impact of clinical sensitivity and specificity and how budget impact results could vary depending on whether test performance is incorporated in the analysis.

We believe that conclusions drawn from this study are largely generalizable to those settings with similar *EGFR* mutation prevalence; however, the magnitude of estimated budget impact or cost savings reported is not generalizable and is dependent on local costs. The results of the base-case analysis are specifically attributed to the test performance data from the cobas^®^ *EGFR* Mutation Test v2. As shown in sensitivity and scenario analyses, test performance is an influential parameter that determines the number of patients with correctly classified *EGFR* mutation status and associated costs; however, lowering the plasma test performance estimates for T790M mutation detection did not change conclusions about the optimal approach for implementing *EGFR* liquid biopsy in the 2L setting. We recognize that, in certain settings, next generation sequencing (NGS) has been implemented into routine clinical practice. For these settings, the research question differs as the interest may be whether it is cost-effective to use a large sequencing panel to simultaneously test multiple genes versus a few individual genes for which targeted therapies are available. Our study is relevant to those settings where NGS may not be available and where molecular diagnostics are primarily used to guide therapy decisions, with the research question of interest being whether there are clinical and budget impact differences between different *EGFR* mutation testing approaches and specimen types. To our knowledge, this is the first study to evaluate the financial impact of adopting an *EGFR* mutation plasma test in the Greek National Health System. In-line with our findings, Gancitano et al. [39] conducted a similar cost-consequence analysis from the National Italian Health System perspective and found that the use of plasma testing strategies identified more *EGFR* mutation-positive mNSCLC patients eligible for targeted therapy with lower average diagnostic costs relative to tissue testing alone.

## 5. Conclusions

These findings demonstrate that access to both *EGFR* mutation tissue and plasma testing are important for optimizing guideline-recommended mNSCLC treatment decisions in Greece. Adopting *EGFR* mutation plasma testing in a combined or reflex testing approach in the 1L setting and a reflex testing approach in the 2L setting enabled more patients to undergo molecular diagnostic testing and identified more *EGFR* mutation-positive patients eligible for targeted therapy compared with tissue testing or plasma testing alone. In addition to the clinical benefits of liquid biopsy, implementing *EGFR* mutation plasma testing via a combined or reflex testing approach may also yield cost savings to the Greek health system through avoiding risks and costs associated with invasive lung tumor tissue biopsies.

## Figures and Tables

**Table 1:** Epidemiological estimates used to project eligible diagnostic test population.

Parameter	Estimate	Reference
Greece population per last census report (2018)	10,741,165	[25]
Lung cancer incidence	7.5/10,000	[26]
% lung cancer cases that are non-small cell (NSCLC)	87%	[3]
% NSCLC that are adenocarcinoma, large cell, or unspecified histology	55%	[3]
% NSCLC advanced or metastatic at diagnosis (stage IIIb or IV)	80%	[3]
**First-line setting (1L)**		
% metastatic NSCLC patients eligible for treatment upon diagnosis (1L)	90%	Clinical opinion
% 1L patients eligible for lung tissue biopsy	85%	[27]
% 1L patients eligible for plasma test	100%	Assumption
*EGFR* mutation prevalence (exons 18 through 21)	15.7%	[5]
**Second-line setting (2L)**		
% mNSCLC patients eligible for treatment upon tumor progression (2L)	50%	[28]
% 2L patients eligible for lung tissue re-biopsy	80%	[18]
% 2L patients eligible for plasma test	100%	Assumption
*EGFR* T790M mutation prevalence	56%	[29,30,31]

**Table 2:** Clinical inputs used in the base-case analysis.

Parameter	Estimate	Reference
Sensitivity: Tissue (exons 19 and 21)	98.1%	[32]
Specificity: Tissue (exons 19 and 21)	99.3%	[32]
Sensitivity: Tissue (T790M)	88.3%	[33]
Specificity: Tissue (T790M)	97.3%	[33]
Sensitivity: Plasma (exons 19 and 21)	94.0%	[33]
Specificity: Plasma (exons 19 and 21)	94.0%	[33]
Sensitivity: Plasma (T790M)	93.0%	[33]
Specificity: Plasma (T790M)	92.0%	[33]
Probability of adverse event or tissue biopsy complication	11%	[20,21]

**Table 3:** Cost inputs used in the base-case analysis.

Parameter	Cost (€)	Reference
***EGFR* Mutation Tissue Test**		
Specimen collection/lung tissue biopsy (CT-guided aspiration)	95	[35]
Treat biopsy complication (assumed severe pneumothorax)	566	[36]
*EGFR* mutation test (procedural reimbursement)	160	[37]
***EGFR* Mutation Plasma Test**		
Specimen collection/blood draw	0	Blood draw procedure is not separately reimbursed
*EGFR* mutation test (procedural reimbursement)	160	Assumed same as *EGFR* mutation test with tissue specimen

**Table 4:** Results of Base-Case Analysis.

Results	Tissue Test Only	Plasma Test Only	Combined Testing Approach	Reflex Testing Approach
Newly Diagnosed mNSCLC Patients in Greece (2018)	3084			
**EGFR Mutation Testing for 1L Therapy (exons 19 and 21)**				
Projected Number of Patients Eligible for EGFR Mutation Testing	2359	2775	2775	2775
Number of Patients with Correctly Classified EGFR Mutation Status	2338	2609	2730	2749
Number of Identified EGFR Mutation Positive Patients	363	410	425	431
Total Cost of Testing Approach	€670,597	€444,066	€737,207	€981,777
BI per Correctly Classified Patient (projected scenario—current scenario)	Current Scenario	−€117	−€17	€70
BI per mNSCLC Patient (projected scenario–current scenario)	Current Scenario	−€73	€22	€101
**EGFR Mutation Testing for 2L Therapy (T790M)**				
Projected Number of Patients Eligible for EGFR Mutation Testing	1234	1542	1542	1542
Number of Patients with Correctly Classified EGFR Mutation Status	1138	1463	1424	1512
Number of Identified EGFR Mutation Positive Patients	610	803	771	846
Total Cost of Testing Approach	€350,639	€246,703	€399,980	€402,387
BI per Correctly Classified Patient (projected scenario—current scenario)	Current Scenario	−€139	−€27	−€42
BI per mNSCLC patient (projected scenario–current scenario)	Current Scenario	−€34	€16	€17

**Table 5:** Scenario analysis: the results in the 2L setting with lower plasma test performance (sensitivity 67%, specificity 80%).

Results	Tissue Test Only	Plasma Test Only	Combined Testing Approach	Reflex Testing Approach
***EGFR* Mutation Testing for 2L Therapy (T790M)** **(Plasma test sensitivity 67%; specificity 80%)**				
Projected Number of Patients Eligible for *EGFR* Mutation Testing	1234	1542	1542	1542
Number of Patients with Correctly Classified *EGFR* Mutation Status	1138	1239	1363	1428
Number of Identified *EGFR* Mutation Positive Patients	610	579	726	780
Total Cost of Testing Approach	€350,639	€246,703	€399,980	€434,926
BI per Correctly Classified Patient (projected scenario—current scenario)	Current Scenario	−€109	−€15	−€4
BI per mNSCLC patient (projected scenario—current scenario)	Current Scenario	−€34	€16	€27
